# Supplementary material for: Effectiveness of a modified Balint group process on empathy and psychological skills employing Kirkpatrick’s evaluation framework
Source: PeerJ. 2023 Jul 19;11:e15279. doi: 10.7717/peerj.15279 (PMC10362841; doi:10.7717/peerj.15279)
Supplement: Supplemental Information 3 [file peerj-11-15279-s003.docx]

Appendix A: Focus group interview guide

The first Focus group

Thank you all for agreeing to participate. This focus group is intended to know your feedback on the Balint session.

The information you give is completely confidential and will not association your name with anything you say in the focus group.

We would like to tape the focus groups so that we can make sure to capture the thoughts, ideas and opinions. No names will be attached and the tapes will be destroyed as soon as they are transcribed.

We ask everyone to respect each other and please refrain from stating any sensitive information ro any information that may identify specific incidents/individuals.

You may refuse to answer any questions or withdraw from the study at any time.

Main questions are in bold; probing questions are in italic.

1. **How satisfied are you with the Balint sessions?** Do you know the purpose behind these sessions? Does it bring you any anxiety? Are you finding what you have in mind?
2. **What part of the Balint session was the most beneficial?** Did you find the debriefing beneficial?
3. **Has participating in the Balint seminar caused you to rethink in some way your role as family doctor or professional identity as physician?** The way you see your relationship with your patient.
4. **How did the Balint seminar affect your psychological skills?** How do you describe your interest, confidence, and clinical abilities in addressing the psychological aspects of patient care?
5. **How do you evaluate the Balint seminar experience as a whole?** Do you think it is important to be part of other residency programs as well? What are your suggestions to make it more effective?

The second focus group

This focus group is intended to know your feedback on the Balint session.

The information you give is completely confidential and will not association your name with anything you say in the focus group.

We would like to tape the focus groups so that we can make sure to capture the thoughts, ideas and opinions. No names will be attached and the tapes will be destroyed as soon as they are transcribed.

We ask everyone to respect each other and please refrain from stating any sensitive information ro any information that may identify specific incidents/individuals.

You may refuse to answer any questions or withdraw from the study at any time.

Main questions are in bold; probing questions are in italic.

1. **How satisfied are you with the Balint sessions SINCE the modification?** Do you know the purpose behind these sessions? Does it bring you any anxiety? Are you finding what you have in mind?
2. **What part of the Balint session was the most beneficial SINCE the modification?**
3. **Has participating in the Balint seminar caused you to rethink in some way your role as family doctor or professional identity as physician SINCE the modifications?** The way you see your relationship with your patient.
4. **How did the Balint seminar affect your psychological skills  SINCE the modifications?** How do you describe your interest, confidence, and clinical abilities in addressing the psychological aspects of patient care?
5. **How do you evaluate the Balint seminar experience as a whole  SINCE the modifications?** Do you think it is important to be part of other residency programs as well? What are your suggestions to make it more effective?
6. **Did you notice a difference in the Balint process since the modification? is it more helpful or more confusing? In what way?**
