# Supplementary figures and images for: Effectiveness of a modified Balint group process on empathy and psychological skills employing Kirkpatrick’s evaluation framework
Source: PeerJ. 2023 Jul 19;11:e15279. doi: 10.7717/peerj.15279 (PMC10362841; doi:10.7717/peerj.15279)

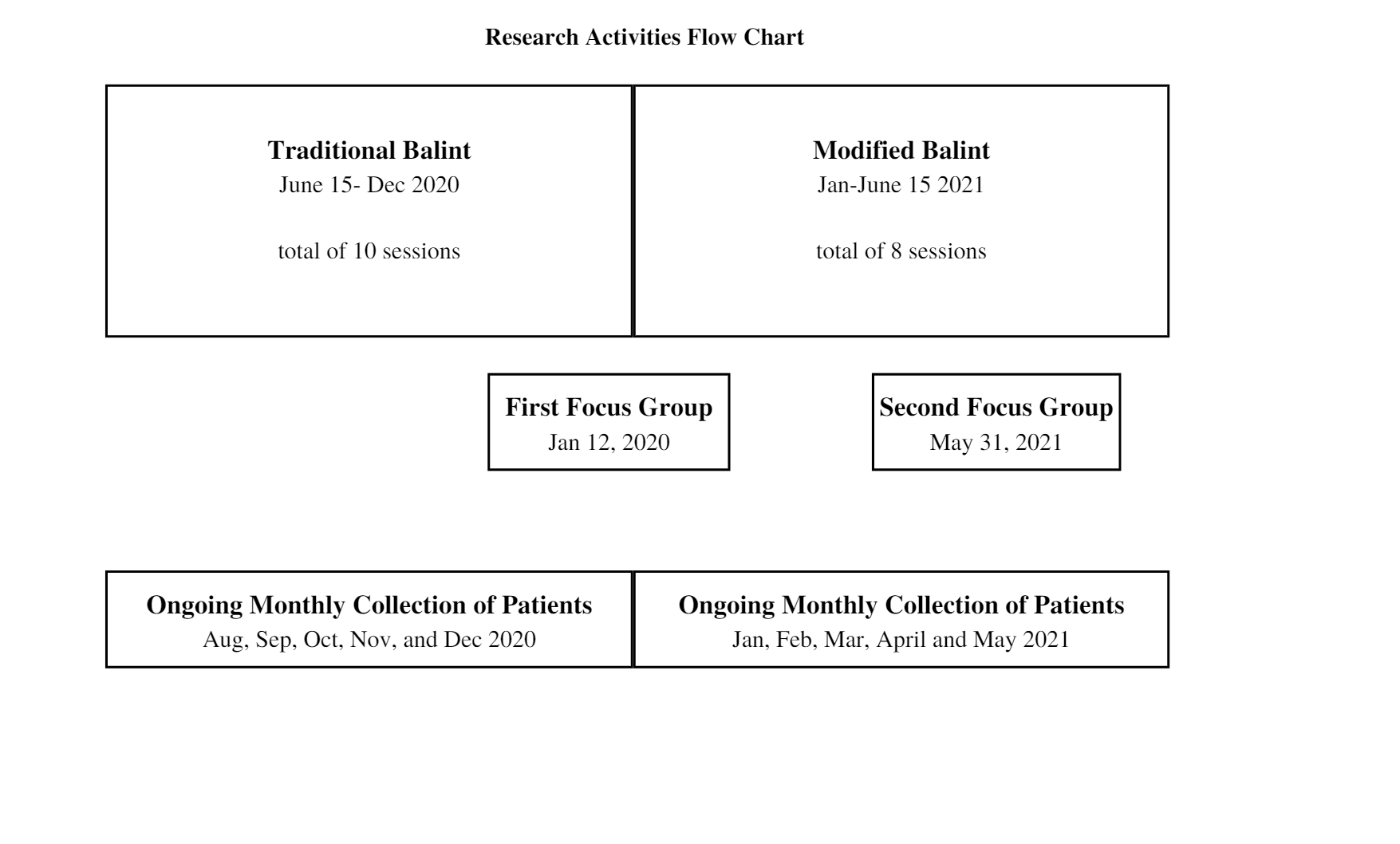

Supplement: Supplemental Information 4 [file peerj-11-15279-s004.png]
